# Satisfaction with Health Services among the Citizens of Serbia

**DOI:** 10.3389/fphar.2017.00050

**Published:** 2017-02-09

**Authors:** Natasa M. Mihailovic, Sanja S. Kocic, Goran Trajkovic, Mihajlo Jakovljevic

**Affiliations:** ^1^Department of Social Medicine, Institute of Public Health KragujevacKragujevac, Serbia; ^2^Department of Social Medicine, Faculty of Medical Sciences, University of KragujevacKragujevac, Serbia; ^3^Faculty of Medicine, Institute for Medical Statistics and Informatics, University of BelgradeBelgrade, Serbia; ^4^Health Economics and Pharmacoeconomics, Faculty of Medical Sciences, University of KragujevacKragujevac, Serbia

**Keywords:** satisfaction, health care utillitation, inequality, determinants, health systems

## Satisfaction with health services

The health system in Serbia has changed a lot in the past 30 years. After falling apart of Yugoslavia in the 90s, all the weaknesses of the health system of that time have become more visible. The country has entered the period of transition, and the creators of health policies have been forced to start the reforms of the health system related to solving structural and functional issues, human resources issues, financing, organization, and availability of the health care in order to build a generally accepted and maintainable health system which shall include intensive controls of the expenses (Kutney-Lee et al., 2009; Kutzin et al., 2010; Jakovljevic et al., 2011; Jakovljevic, 2013).

Following democratic changes during 2000, Serbia has entered the period of intensive reforms and speedy recovery of the health system, due to the significant income of money through International charity help and affordable bank loans (Bajec et al., 2008). Quick development was stopped by the world economic crisis in 2007 (Marmot et al., 2013; Ruckert and Labonté, 2014; Tøge and Blekesaune, 2015). Today, Serbian government provides the health system with 10.4% of Gross domestic product (GDP), and alongside with Belgium, Denmark, and Canada, Serbia falls under the category of countries which provides health system with a significant part of GDP, but that percentage is considerably smaller than in the countries already mentioned (The World Bank, 2016).

The reform analysis of health systems going through a transition and the analysis of quality assessment of the provided services are conducted using the indicators for the subjective and objective assessment. The most common indicators for the subjective assessment are: the satisfaction of the patient with health care and self-assessment of a health condition. The satisfaction with health care is used in the reform analysis of the health systems all across Europe, Asia and America and it represents the ratio between the expected and achieved health services (Bleich et al., 2009; Browne et al., 2010; Rechel et al., 2012; Gupta et al., 2015).

The most common determinants of the satisfaction of the citizens with health care are: age, health condition, income, the type of the given service (state or private sector), communication, politeness of the staff, and hospital environment (Friese et al., 2008; Aiken et al., 2011, 2012; Al-Refaie, 2011; Xesfingi and Vozikis, 2016).

Due to the lack of universal instrument for measuring the level of satisfaction with health care and the lack of correlation of the satisfaction and economic power of the health system, the degree of satisfaction with health services is determined indirectly based on: waiting lists, quality of given services and communication with health workers (Sofaer and Firminger, 2005; Adang and Borm, 2007).

## The data report methods

### Public data set description—National Health survey Serbia 2006 and 2013

The research has used the database of the two National Health Surveys in the Republic of Serbia, conducted in 2006 and 2013, funded by the Ministry of Health (National health survey Serbia, 2006, 2013). The survey was conducted in accordance with the methodology and instruments of the European Health Interview Survey wave 2 (Eurostat European Commission, 2013). Both surveys were conducted as cross sectional studies. Population presented in the research included adults, aged 19, and more. The researches excluded people living on the territory of Kosovo and Metohija, as well as people with residence addresses in Special institutions (retirement homes, prisons, psychiatric clinics).

The research used the national representation sample, such as stratified two-phase sample without repetition. The sample frame in the 2006 and 2013 researches included all the households listed in the censuses from 2002 to 2011. In order to obtain a random sample, two techniques have been used: stratification and multiple-phase sampling. Stratification was conducted in such a way that each of six geographical areas in 2006 (Vojvodina, Belgrade, west Serbia, central Serbia, east Serbia, and south-east Serbia) and 4 in 2013 (Vojvodina, Belgrade, Sumadija, and west Serbia, south and east Serbia) represented one main stratum in the sample. Each stratum was divided into cities and other regions. Total number of strata was 12 in 2006 and 8 in 2013. Based on such sample, it is possible to obtain a statistically accurate assessment of the health service quality on a national level as well as on the level of given geographical regions and on the level of cities and other areas.

Two-phase sampling includes local communities, as units of the first phase, selected on the basis of probability proportional to their size, and households as units of the second phase selected on the basis of linear sampling method with the random start and uniform selection steps. In this way, households are selected with the equal probability of being chosen, without repetition.

Acquired data are then united in the unique database according to the common key and organization principle.

### Survey data description

The number of households which wanted to participate in the research was significantly different in 2006 and 2013. In 2006 out of 7673 contacted households, 6156 agreed to take part in the research, which represents the response rate of 86.5%. In 2013 the response rate was 64.4%, which means that only 6500 households out of 10,089 contacted ones agreed to participate in the research. Total number of the interviewed adults was 30,186. For the purposes of this paper, the sample of 19-year-olds and older was used, which amounts to the total number of 29,485 interviewees, namely 15,563, and 13,992 in 2006 and 2013 respectively.

Two types of questionnaires have been used in the research: the questionnaires about the household which provided information on the characteristics of the household, residence, and members and the face to face interview which provided data on demographic and socio-economic characteristics of the interviewees. Each interviewee was familiar with the type and the purpose of the research and each of them gave their written consent. Ethical standards in the research were in accordance with the international ethical standards as well as with the Serbian legislation.

Dependent variable in the research was the satisfaction of patients with the health service, measured with Likert-type scale.

Independent variables in the research were:

- Basic characteristics of the interviewees (age, gender, financial condition according to well-being index, region). Age is continuous, gender is coded as male, or female, financial condition according to well-being index for the purpose of this paper was coded into three categories: poor, middle class and rich and the listed regions were Vojvodina, Belgrade, Sumadija with West Serbia and South and East Serbia;
- Health condition of the interviewees (presence of some illness or certain pathological conditions in the past 12 months, sick leave). Illnesses or pathological conditions which were diagnosed in the past 12 months included: asthma, chronic bronchitis, chronic obstructive pulmonary disease, emphysema, myocardial infarction, coronary heart disease, hypertension, stroke, osteoarthritis, diabetes, kidney problems, depression, malignant diseases, and interviewees were or were not on a sick leave during the past 12 months;
- Using hospital and non-hospital health care services and prevention check-ups (hospital treatment choosing a GP or pediatrician, using the private practice services, prevention check-ups, lab analysis, and tension measurement in the health institutions). All these variables are dichotomic;
- Unachieved need for health care (lack of some type of health care service due to long waiting lists, financial reasons, long distance of the health institution). Interviewees could or couldn't get the necessary health treatment in the past 12 months due to long waiting lists;

The data set has been submitted in a public repository Figshare and it is a available on: https://figshare.com/s/b1ede3b1bbcdf166eac7.

### Satisfaction with Health Services in Serbia

During the analyzed period, the percentage of the interviewees satisfied with health care rose from 42.8 to 54.8%. Both with men and women, there is a noticeable increase of the satisfaction level and the satisfied patients were 1 year older in 2013 in comparison to 2006 (52.27 ± 17.45, in comparison to 53.86 ± 17.98, *p* < 0.001). The inhabitants of the region of Sumadija and West Serbia were the most satisfied in 2006, while in 2013 the people of Vojvodina were the most satisfied ones. The least satisfied were the rich, namely 39.2% in 2006 and 48.1% in 2013. The interviewees which were diagnosed with some disease in the past 12 months were equally satisfied as those who were not diagnosed with any disease. Patients who were on sick leave in the past 12 months were less satisfied while the difference in satisfaction between hospitalized and not hospitalized patients which was noticed in 2006 (55.6% of hospitalized as opposed to 41.9% not hospitalized) faded away and the percentages became identical in 2013. The biggest difference in patient satisfaction (apart from the variable of unachieved need) was recorded in choosing a general practitioner. In 2006, 50% of the interviewees had their own GP and one third of the total number of the interviewees were satisfied. The percentage of those who used the private practice services and who were satisfied with them was 34.7 and 41.4% in 2006 and 2013 respectively, and the percentage of those who had preventive check-ups in the last 12 months was 41.6 and 54.6% respectively. Almost half of the total number of interviewees in 2006 expressed their satisfaction although they did not get the necessary treatment on time. On the other hand, in 2013 only one quarter of the total number of the interviewees who did not get the necessary treatment on time, were satisfied with the provided services (Table 1).

**Table 1:** **The comparison of the patients who were satisfied with health care in 2006 and 2013**.

**Variables**	**Year 2006**		**Year 2013**		***p***
	**Total**	n (%)^*^	**Total**	n (%)^*^	
Total	15563	6119 (42.8)	13922	7154 (54.2)	<0.001
**GENDER**					
Female	7586	3526 (46.5)	7276	4032 (55.4)	>0.05
Male	6703	2593 (38.7)	5927	3122 (52.7)	
**REGION**					
South and East Serbia	3667	1587 (43.3)	3211	1752 (54.6)	<0.001
Vojvodina	3555	1321 (37.2)	3218	1617 (50.2)	
Belgrade	2559	1049 (41)	2724	1409 (51.7)	
Sumadija and West Serbia	4508	2162 (48)	4050	2376 (58.7)	
**WELL-BEING INDEX**					
Poor	3176	1365 (43)	2947	1741 (59.1)	<0.05
Middle class	8661	3792 (43.8)	7964	4336 (54.4)	
Rich	2452	962 (39.2)	2292	1077 (47)	
**ILLNESSES IN THE PAST 12 MONTHS**					
No	11881	5096 (42.9)	6139	3246 (52.9)	<0.001
Yes	2408	1023 (42.5)	7064	3908 (55.3)	
**SICK LEAVE**					
No	14056	6030 (42.9)	3588	1782 (49.7)	<0.001
Yes	233	89 (38.2)	652	250 (38.3)	
**HOSPITALIZATION**					
Yes	959	533 (55.6)	1179	664 (56.3)	>0.05
No	13258	5555 (41.9)	12024	6490 (54)	
**SELECTED GP**					
No	7113	3593 (50.5)	937	356 (38)	<0.001
Yes	7166	2522 (35.2)	12250	6792 (55.4)	
**PRIVATE PRACTICE**					
No	11631	5196 (44.7)	11112	6292 (56.6)	<0.001
Yes	2645	918 (34.7)	2091	862 (41.2)	
**PREVENTIVE CHECK-UPS**					
More than 12 months ago	5105	2301 (45.1)	4486	2430 (54.2)	<0.001
In the last 12 months	9145	3803 (41.6)	8672	4699 (54.2)	
**UNACHIEVED NEED**					
No	11897	5017 (42.2)	11413	6665 (58.4)	<0.001
Yes	2378	1094 (46)	1751	473 (27)	

n (%)

* *, the number (percentage) of whose satisfied with health care*.

The results of the binary logistic regression of the patient satisfaction with health care in the period from 2006 to 2013 show that the following variables give a statistically significant contribution to the model: age, illnesses diagnosed in the past 12 months, hospitalization, selected GP, using the private practice services and preventive check-ups in the past 12 months. In 2006, there were more statistically significant predictor variables: region and well-being index, and in 2013 there was sick leave and unachieved need for some type of health care (Table 2).

**Table 2:** **Binary logistic regression for Health Services Satisfaction in 2006 and 2013**.

**Variables**	**Year 2006**		**Year 2013**	
	**OR (95% CI)**	***p***	**OR (95% CI)**	***p***
Age	0.992 (0.990–0.994)	<0.001	0.993 (0.987–1.000)	<0.05
**REGION**				
South and East Serbia	1		1	
Vojvodina	1.337 (1.213–1.475)	<0.001	1.141 (0.944–1.379)	>0.05
Belgrade	1.293 (1.153–1.449)	<0.001	0.878 (0.722–1.066)	>0.05
Sumadija and West Serbia	0.0804 (0.734–0.880)	<0.001	0.917 (0.769–1.094)	>0.05
**WELL-BEING INDEX**				
Poor	1		1	
Middle class	0.900 (0.825–0.982)	<0.05	0.985 (0.802–1.208)	>0.05
Rich	0.994 (0.880–1.122)	>0.05	1.123 (0.888–1.421)	>0.05
**ILLNESSES IN THE PAST 12 MONTHS**				
No	1		1	
Yes	1.212 (1.102–1.333)	<0.001	1.182 (1.027–1.361)	<0.05
**SICK LEAVE**				
No	1		1	
Yes	1.249 (0.948–1.646)	>0.05	1.390 (1.141–1.694)	<0.05
**HOSPITALIZATION**				
Yes	1		1	
No	1.551 (1.349–1.783)	<0.001	1.465 (1.070–2.006)	<0.05
**SELECTED GP**				
No	1		1	
Yes	2.010 (1.867–2.164)	<0.001	0.506 (0.391–0.655)	<0.001
**PRIVATE PRACTICE**				
No	1		1	
Yes	1.556 (1.417–1.708)	<0.001	1.626 (1.359–1.944)	<0.001
**PREVENTIVE CHECK-UPS**				
More than 12 months ago	1		1	
In the past 12 months	1.124 (1.047–1.207)	<0.05	1.199 (1.055–1.364)	<0.05
**UNACHIEVED NEEDS**				
No	1		1	
Yes	0.914 (0.833–1.003)	>0.05	4.576 (3.553–5.892)	<0.001

The older the interviewees were, the less satisfied they were. Odds ratio with patients who were diagnosed with some illness was 1212 (1102–1333) in 2006 and 1182 (1027–1361) in 2013. Patients who were not hospitalized in the last 12 months were 1.5 or 1.4 times more dissatisfied with health care in comparison with those who were hospitalized. Less satisfied were those who had preventive check-ups in the last 12 months with those who did not. The biggest difference was recorded with the predictor variable of the selected GP, namely *OR* = 2.010 (1867–2164) in 2006 and in 2013 *OR* = 0506 (0391–0655). The interviewees who used private practice services were 1.5 and 1.6 times less satisfied in comparison to those who did not use them.

Based on the research from 2006, people coming from Vojvodina and Belgrade were 1.3 and 1.2 times less satisfied in comparison to the people from South and East Serbia.

Based on the research from 2013, patients who were on sick leave had *OR* = 1390, which means that they were 1.3 times less satisfied in comparison to the interviewees who were not absent from work. The strongest predictor of patient satisfaction with health care was unachieved need for some type of health protection. Namely, in 2013 patients who did not get the necessary treatment were Four times less satisfied with health care in comparison to those who did not have such problems (*OR* = 4579 (3553–5892).

### In comparison to the world

The analysis of the patient satisfaction with the health protection services has a goal of improving the quality of the given services, defining priorities, understanding patients' expectations and reducing inequality (Chow et al., 2009; Gupta et al., 2014; Radevic et al., 2016) The researches of the patient satisfaction on a national level enable monitoring of trends, defining problems, analyzing predictors for certain population groups in relation to gender, age, the type of the provided service and the type of the medical institution (U. S. Department of Health Human Services., 2011; Republic of Serbia Ministry of health, 2014). Regardless of possibilities, patient satisfaction with health protection, as a subjective indicator, hasn't still been used in its full scope (Jenkinson et al., 2002; Al-Abri and Al-Balushi, 2014).

In the period between 2006 and 2013, the population in the Republic of Serbia reduced for 3.3% (247,437). In the same time, life expectancy increased during the period of about 3 years (it rose from the average 72,37–75,05 years of age for both genders; Institute of Public health of Serbia, 2007, 2014). Health care expenses, by looking at GDP, rose from 9 to 10.4% of GDP (The World Bank, 2016). However, patient satisfaction as a subjective factor does not have to correlate with the budget and real performance of the health system (Adang and Borm, 2007). Due to the influence of the transition and the related socio-economic changes on patient satisfaction with health care, they have been used in an analysis of relations and attitudes of patients toward the reforms of the health system (Footman et al., 2013). The analysis of the quality indicators related to medical services shows that the satisfaction has significantly decreased in the countries going through a period of transition, especially following the world economic crisis in 2007 (Habibov and Afandi, 2015). Although inequality in using medical services is constantly present (Abebe et al., 2016), there are certain differences related to the period before and after 2007. So the number of those people who consider their health worse after the world economic crisis increased among the inhabitants of Greece, Lithuania, Poland, and Estonia (Vandoros et al., 2013; Zavras et al., 2013; Hessel et al., 2014; Reile et al., 2014).

The analysis conducted in the countries of West and East Europe shows diverse results. The research conducted in six countries of Central and East Europe, shows that there were 10–14% dissatisfied patients. However, some differences could be noticed, namely, patients from Hungary were the most satisfied while the most dissatisfied patients came from Bulgaria and Ukraine (Stepurko et al., 2016). However, the research of patient satisfaction trends conducted in the Netherlands in the period between 2003 and 2009 showed opposing arguments. Namely, according to one group this period recorded a global rise of patient satisfaction while the others argue that the satisfaction among hospitalized patients decreased from 76 to 66% (Kleefstra et al., 2015).

Patient satisfaction is related to the positive results of treatments, and such patients are easier to treat although they often ask for additional services, but researches show that satisfaction remains even when the additional requests have not been met (Zolnierek and Dimatteo, 2009; Deyo et al., 2010; ACCORD Study Group et al., 2011; Bertakis and Azari, 2011; Wiener et al., 2011). The research of satisfaction factors shows that the communication with medical workers, along with employment status, education and gender is one of the most important factors influencing patient satisfaction. Moreover, women, less educated people, and unemployed people estimate their own health condition as worse (Zolnierek and Dimatteo, 2009; Sánchez-Piedra et al., 2014).

Similar to our research, there was a research conducted in nine countries of the former SSSR which showed increased patient satisfaction with health care from 19.4% at the beginning to 40.6% in the period between 2001 and 2010. Similarly to our country, these countries also recorded that the most satisfied patients were young people, less educated people, people coming from rural places, and those people with generally good health condition (Footman et al., 2013). The analysis of the type of medical services shows that people who rarely use services of the Emergency center and, as in our research, those people who often use hospital services were more satisfied (Fenton et al., 2012). It is interesting that there is no significant relation between the satisfaction of hospitalized patients and hospitalization rate while nursing care, medical care and hospital organization are the most important factors determining the level of satisfaction among hospitalized patients in Germany (Sacks et al., 2015; Kraska et al., 2016).

## Conclusive remarks

Patient satisfaction with health care represents the path toward the improvement of its quality. However, this instrument is most often used in order to define the factors which determine the satisfaction. In order to use it as means of monitoring and improving the quality, it is necessary to acquire additional knowledge about specific aspects of patient experience in relation to their characteristics, but also about the type and characteristics of the health institution which provided the services as well as the type of the provided services.

## Author contributions

All authors listed, have made substantial, direct, and intellectual contribution to the work, and approved it for publication. NM: drafting of manuscript; SK: interpretation of data; GT: analysis of data; MJ: conception and design of the manuscript.

### Conflict of interest statement

The authors declare that the research was conducted in the absence of any commercial or financial relationships that could be construed as a potential conflict of interest.
